# Evaluation of KIF23 variant 1 expression and relevance as a novel prognostic factor in patients with hepatocellular carcinoma

**DOI:** 10.1186/s12885-015-1987-1

**Published:** 2015-12-16

**Authors:** Xiaotong Sun, Zhongtian Jin, Xiao Song, Jingjing Wang, Yan Li, Xiaoping Qian, Yu zhang, Yanhui Yin

**Affiliations:** 1 Department of Immunology, School of Basic Medical Sciences, Peking University Health Science Center, 38 Xueyuan Road, Haidian District, Beijing, 100191 China; 2 Center of Hepatobiliary Surgery, People’s Hospital, Peking University Health Science Center, Beijing, China

**Keywords:** KIF23, Hepatocellular carcinoma, Immunohistochemisty, Overall survival, Prognostic factor

## Abstract

**Background:**

KIF23 (kinesin family member 23) is a kinesin-like motor protein and plays an important role in cytokinesis. In search for genes associated with hepatocellular carcinoma (HCC) by cDNA microarray, we found that KIF23 was upregulated in HCC tissues. At present, much less is known about its expression and functions in tumor cells. In this work, we aimed to investigate the expression of KIF23 in HCC and the correlation between its expression and clinical features.

**Methods:**

Total RNA was extracted from 16 HCC and paired adjacent non-cancerous tissues. The expressions of the two KIF23 splice variants (KIF23 V1 and KIF23 V2) in normal and HCC tissues were determined by reverse transcriptase polymerase chain reaction (RT-PCR). Polyclonal antibody specific to KIF23 V1 was prepared, and the specificity of the antibody was confirmed by siRNA knockdown and Western blotting experiments. KIF23 protein expression in HCC was examined by immunohistochemistry staining with anti-KIF23 V1 or anti-KIF23 (commercially available for recognizing both KIF23 V1 and V2) antibodies, respectively. Univariate and Multivariate Cox regression analyses were used to determine the correlation between KIF23 protein expression and overall survival of HCC patients.

**Results:**

The two splicing variants of KIF23 mRNA were not detected in normal liver tissue by RT-PCR, but they were aberrantly expressed in HCC tissues. Immunohistochemistry staining with anti-KIF23 V1 antibody revealed that KIF23 V1 was mainly distributed in the nucleus, whereas the positive staining signals were predominantly in the cytoplasm when using anti-KIF23 antibody, suggesting that KIF23 V2 might localize in the cytoplasm of HCC cells. KIF23 V1 protein was detected in 57.6 % (83/144) HCC patients and the mean *H*-score was 42, while KIF23 V2 was detected in 94.4 % (135/143) HCC samples and the mean *H*-score was 68. Follow-up study showed that HCC patients with expression of KIF23 V1 had a longer 5-year survival (*p* = 0.0052), however, expression of KIF23 V2 protein did not associate with 3- and 5-year survival.

**Conclusion:**

In this study we show for the first time that KIF23 V1 and V2 have different localizations in HCC cells. Furthermore, KIF23 V1 protein expression might be a marker of longer overall survival in HCC patients.

## Background

Hepatocellular carcinoma (HCC), the major type of primary liver cancer, is one of the most prevalent cancers in the world [1]. China is one of the high-risk areas for HCC due to the high prevalence of chronic hepatitis B virus infection [2, 3], representing more than half of the cases in the entire world [4]. Despite the remarkable advances in diagnostic and therapeutic techniques, prognosis of HCC still remains extremely poor, ranking as the third leading cause of cancer-related death worldwide [1]. Therefore, numerous studies have focused on screening for novel diagnostic and prognostic biomarkers as well as therapeutic targets in HCC [5–7].

We have performed cDNA microarray analysis for mining differentially expressed genes in HCC in an attempt to identify new HCC biomarkers [5]. Assessing microarray data, we found that the expression of KIF23 showed a 6-fold increase in HCC tissues compared with paired non-cancerous tissues. KIF23, also known as CHO1/MKLP1, was first identified as a motor enzyme that moves antiparallel microtubules in vitro [8]. Subsequent studies indicated that KIF23 is a key regulator of cytokinesis [9, 10]. The disfunction of KIF23 resulted in incomplete cytokinesis and formed binucleated or multinucleated cells [11, 12], which have been considered as the hallmarks of the cancer cells [13]. However, to date, only few studies have been reported on the expressions of KIF23 in tumor cells. Valk K et al. found that KIF23 is upregulated in non-small cell lung cancer (NSCLC) in screening differentially expressed genes in NSCLC [14]. Recently, Takahashi S et al. reported a higher level of KIF23 expression in glioma tissues compared to normal brain tissue [15]. However, the expression of KIF23 in HCC tissues remains unknown.

Human KIF23 has two splice variants, KIF23 V1 and KIF23 V2. When we use the term KIF23, it refers to both KIF23 V1 and V2. KIF23 V1 is different from KIF23 V2 only in that it contains an extra 312 bp sequence (exon 18) in the COOH-terminal tail [16]. Intriguingly, the sequence encoded by exon 18 is an F-actin interacting domain, which may be essential for special functions of KIF23 V1. However, all the commercial anti-KIF23 antibodies recognize both KIF23V1 and V2, thus little is known about the expressions and functions of each individual variant in tumor cells so far. Since the nucleotide sequence encoding KIF23 V2 is completely same as the sequence for KIF23 V1 except lacking the exon 18, it is difficult to investigate KIF23 V2 alone. Thus we generated anti-KIF23 V1 antibody, and detected the expressions of the two isoforms of KIF23 in HCC samples with antibodies specific for KIF23 V1 or for both KIF23 V1 and V2 by immunohistochemistry. We also investigated the prognostic significance of the expressions of KIF23 V1 and KIF23 V2 on overall survival of HCC patients.

## Methods

### Patients and samples

Ninety-eight HCC patients who underwent partial hepatectomy or liver transplantation in the Peking University People’s Hospital, and commercial microarrays consisting of 46 HCC patients (Shanghai Outdo Biotech, China) were enrolled in this study for immunohistochemical analysis. In each case, the HCC diagnosis was confirmed by post-operative pathological examination. Written informed consent was obtained from all participating patients, according to our university guidelines. The study included 120 males and 24 females aged between 32 and 85 years with a median age of 55 years. All the patients were classified according to the 6th edition of the TNM classification of the International Union Against Cancer, and there are 42 patients with stage I, 34 patients with stage II, 46 patients with stage III, and 22 patients with stage IV. Among the studied 144 patients, there are 80 patients with tumor size more than 5 cm. Follow-up data were not available for 32 patients leaving 102 patients for final evaluation of survival. Overall survival (OS) was defined as the time between surgery and death of any cause or last follow-up.

Fourteen different normal tissue cDNA preparations, including heart, placenta, lung, liver, skeletal muscle, kidney, pancrease, spleen, thymus, prostate, ovary, small intestine, colon, and peripheral blood leukocyte, were purchased from Clontech Laboratories Inc. Sixteen pairs of frozen tumor tissues and adjacent non-tumor tissues for detection of KIF23 mRNA expression were from the Peking University People’s Hospital.

The experiment was conducted in compliance with the Helsinki declaration and was approved by the Ethics Review Committee of Peking University of Health Science Center.

### Cell lines, plasmids and siRNAs

The human cell lines HLE, Huh7, HepG2, SMMC-7721, BEL-7402 and HEK293T cells were grown in DMEM containing 10 % fetal calf serum (FCS). Transient transfection of plasmid constructs was performed using Lipofactamine 2000 (Invitrogen) in HEK293T cells, and siRNAs were transfected using jetPRIME (Polyplus transfection) in HLE cells. To construct pRK-FLAG-KIF23 V1 and pRK-FLAG-KIF23 V2 expression vectors, human KIF23 V1 and V2 cDNA fragments amplified by reverse transcription-PCR (RT-PCR) from HLE cell RNA were cloned into the pRK-FLAG vectors, respectively. KIF23 V1 siRNA1 (5’-GUACAACACACCUCUCAAATT-3’) (specific for KIF23 V1), KIF23 V1 siRNA2 (5’- GCAGUCUUCCAGGUCAUCUTT-3’) (target both KIF23 V1 and V2), and control siRNA (5’–UUCUCCGAACGUGUCACGUTT-3’) were synthesized by RiboBio Co. Ltd (Guangzhou, China).

### Reverse transcription (RT)-PCR

Total RNA of the tumor and paired adjacent noncancerous tissues was isolated using TRIzol reagent (Invitrogen, USA), and first strand cDNA was generated using random primers and AMV reverse transcriptase (Progema, USA) according to the manufacturer’s instructions. Primer sequences specific for amplifying KIF23 V1 were 5’-CAGATTTCCAACGGCCAGCA-3’ and 5’-TCATGGCTTTTTGCGCTTGG-3’, for amplifying KIF23 V2 were 5’-TCCATCACCTGTGCCTTTACT-3’ and 5’-TGGGACTGTCAGTTCATGGC-3’ (the PCR product is 541 bp for KIF23 V2).

### Whole, cytoplasmic, and nuclear protein extraction

Total cellular protein extracts were obtained using RIPA lysis buffer as described previously [17]. Cytoplasmic and nuclear extracts were prepared as follows: cells were lysed in a hypotonic buffer containing 10 mM pH 7.9 Hepes, 10 mM KCl, 0.1 mM EDTA, 1 mM DTT, 0.15 % NP-40, and protease inhibitor cocktail. After incubating 25 min on ice, samples were centrifuged and supernatants (corresponding to cytoplasmic extracts) were collected. The nuclear pellets were further washed with PBS and then resuspended in RIPA buffer supplemented with protease inhibitor cocktail. After vigorously shaking for 30 min at 4 °C, the nuclear extracts were collected. Whole, cytoplasmic and nuclear extracts were analyzed by Western blotting.

### Western blotting

Equal amounts of cellular extracts were subjected to SDS-PAGE for electrophoresis, transferred to a nitrocellulose membrane, followed by incubation with appropriate antibodies. Tubulin antibody was used to verify equivalent total protein. Immunoreactive bands were visualized with enhanced chemiluminescence or infrared imaging working with Odyssey Imager (Li-Cor, Lincoln, NE).

### Antibodies

The polyclonal anti-KIF23 V1 antibody was generated using a synthesized peptide encompassing the residues 747–761 of the human KIF23 V1 protein (GenBank Accession number: NP_612565), which only presents in KIF23 V1, but not in KIF23 V2 isoform. The peptide conjugated to KLH was used to produce antibody in rabbits. The resultant antibody was purified by immuno-affinity chromatography (GE Healthcare). Anti-KIF23 antibody, recognizing both KIF23 V1 and V2 proteins, was purchased from Santa Cruz (sc867, Santa Cruz), anti-lamin B1 antibody from Bioworld Technology, and anti-tubulin antibody from Sino Biological.

### Immunofluorescence

Cells were grown directly on glass coverslips for 24 h, and then fixed and permeabilized. After blocking in PBS-5 % skimmed milk, cells were incubated with anti-KIF23 V1, and normal rabbit IgG was used as a negative control. After washing with PBS, cells were incubated at room temperature with FITC-conjugated anti-rabbit IgG (Zhongshan company, China). Cell nuclei were stained with Hoechst33342. Images were required using confocal microscope.

### Immunohistochemistry (IHC)

IHC was performed as previously described [18] with minor modifications. Briefly, paraffin-embedded tissue sections were deparaffinized with xylene and rehydrated with a graded series of ethanol. After antigen retrieval, inactivation of endogenous peroxidase, and blocking with normal goat serum, sections were incubated with anti-KIF23 V1 or anti-KIF23 antibodies at 4 °C overnight, followed by adding dextran carrying anti-rabbit IgG conjugated to horseradish peroxidase (HRP) and positive staining was developed using the Dako REAL EnVision detection system. Images of stained sections were imported into Olympus CX31 digital microscope (Olympus, Japan) for quantifying stained cells.

### Evaluation of IHC staining

IHC staining was evaluated by taking into account both the intensity of staining and the percentage of positive cells [19]. Tumor staining intensity was graded on a scale from 0 (negative), 1 (weak), 2 (moderate) to 3 (strong) and each intensity category was scored a percentage of tumor cells ranging from 0 to 100 so that the sum of the percentages adds up to 100. The percentage score was then multiplied by its intensity category to obtain a final *H*-score, ranging from 0 to 300.

### Statistical methods

Statistical analyses were performed using SAS 9.1.3 Portable for Windows (SAS, SAS Institute Inc, USA) and GraphPad Prism 5.0 (GraphPad Software, USA).

The relationships between KIF23 V1 expression and the potential explanatory variables were evaluated with the Chi-square and cmh (Cochran-Mantel-Haenszel) Chi-square tests. The survival rate was analyzed using the Kaplan-Meier method and log-rank test. The univariate examination of the relationship between the assessed criteria and survival was performed with a Chi-square test. A cox proportional-hazard model was used for the multivariate analysis. *P* < 0.05 was considered statistically significant.

## Results

### Expressions of KIF23 V1 and V2 mRNA in normal and HCC tissues

**Fig. 1:**
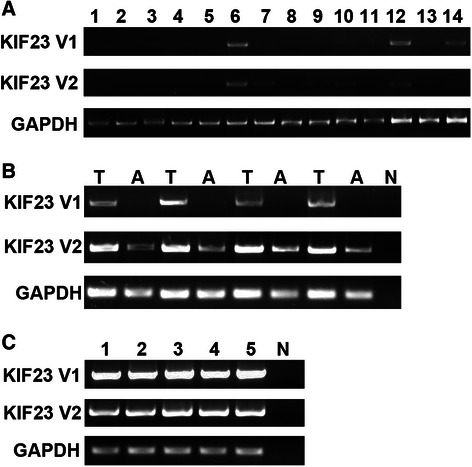
Expressions of KIF23 V1 and KIF23 V2 mRNA in normal and malignant tissues. **a** Expressions of KIF23 V1 and KIF23 V2 mRNAs in normal tissues. Lane 1: heart; 2: liver; 3: skeletal muscle; 4: pancreas; 5: ovary; 6: colon; 7: PBMC; 8: placenta; 9: lung; 10: kindey; 11: spleen; 12: thymus; 13: prostate; 14: small intestine. **b** Representative positive expressions of KIF23 V1 and KIF23 V2 mRNA in some HCC tissues. T: cancerous tissues; A: adjacent noncancerous tissues. **c** Expressions of KIF23 V1 and KIF23 V2 mRNA in HCC cell lines. Lane 1: HLE; 2: SMMC-7721; 3: BEL-7402; 4: Huh7; 5: HepG2; N: negative control

To examine the distribution of mRNA expression of the two splice variants of KIF23, RT-PCR was performed using cDNA reversed from mRNA of a variety of human tissues and human derived cancer cell lines. Both of the two variants were not detected in normal liver tissues (Fig. 1a), but they were found to be aberrantly expressed in HCC tissues (Fig. 1b). KIF23 V1 mRNA was detected in 81.2 % (13/16) of HCC tissues, while V2 mRNA was detected in 100 % (16/16) of HCC tissues. The two variants of KIF23 were all detected in the five HCC cell lines tested (Fig. 1c).

### Generation and characterization of polyclonal antibody specific for KIF23 V1

**Fig. 2:**
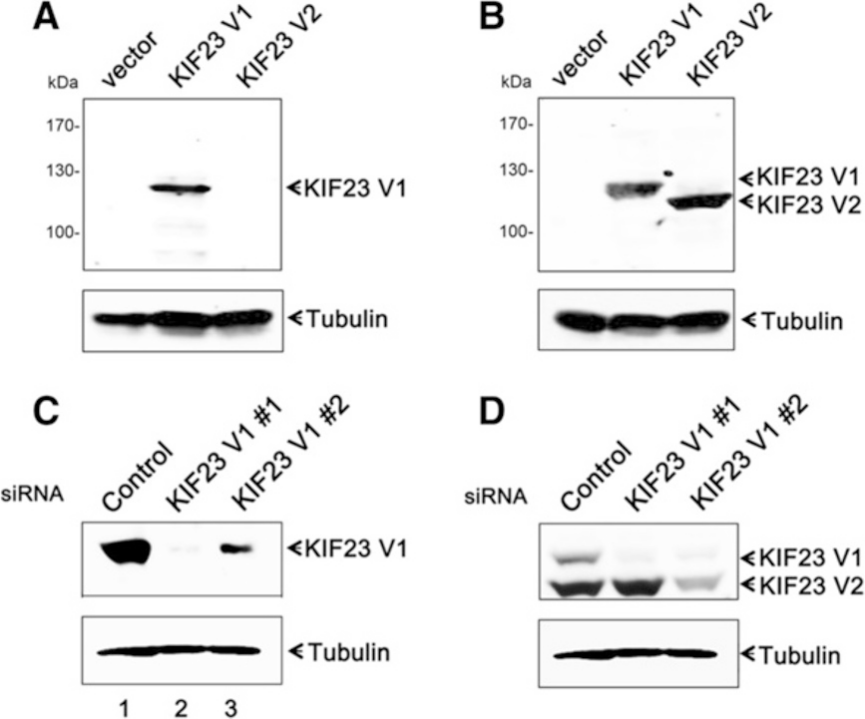
Characterization of anti-KIF23 V1 antibody. **a** HEK293T cells were transiently transfected with pRK-FLAG-KIF23 V1, pRK-FLAG-KIF23 V2, or pRK-FLAG plasmids and the protein expression was detected by Western blotting employing anit-KIF23 V1 antibody. **b** Western blotting analysis of the same samples as in (**a**) was performed with commercial anti-KIF23 antibody. **c** HLE cells were transfected with either control or KIF23 V1 siRNAs and whole cell extracts were processed for immunoblotting with anti-KIF23 V1 antibody. **d** Western blotting analysis of the same samples as in (**c**) was performed with commercial anti-KIF23 antibody. All the membranes were reblotted for the expression of tubulin

To characterize the expression of the two isoforms of KIF23 in HCC, we raised polyclonal antibody directly against the synthetic peptide derived from the sequence unique to the KIF23 V1 isoform. To test whether the antibody can discriminate between KIF23 V1 and its truncated isoform KIF23 V2, plasmids encoding KIF23 V1 or V2 were transiently transfected into human HEK293T cells and total cellular lysates were analyzed by SDS-PAGE followed by immunoblotting with anti-KIF23 V1 or commercial anti-KIF23 antibodies. The anti-KIF23 V1 antibody only detected KIF23 V1 protein, but not KIF23 V2 isoform (Fig. 2a), while both KIF23 V1 and V2 proteins were detected when Western blotting was performed with anti-KIF23 antibody (Fig. 2b).

To test whether the prepared anti-KIF23 V1 antibody can recognize endogenous KIF23 V1 protein within tumor cells, the whole cell extracts of HLE cells was immunoblotted with anti-KIF23 V1 antibody and a single prominent band with expected size was detected (Fig. 2c, lane 1). This band was significantly down regulated in cell extracts derived from KIF23 V1 siRNA-treated HLE cells (Fig. 2c, lane 2, 3), indicating this antibody recognize bona fide KIF 23 V1 protein. This data was supported by the result using commercial anti-KIF23 antibody (Fig. 2d).

### Sublocalization of KIF23 V1 protein in HCC cell lines

**Fig. 3:**
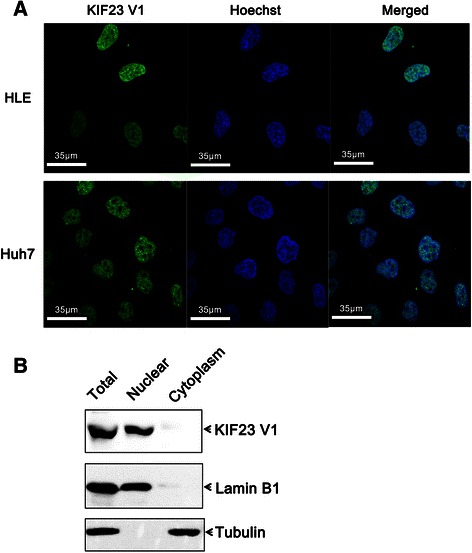
Subcellular distribution of endogenous KIF23 V1 protein. **a** Localization of KIF23 V1 in HLE and Huh7 cells examined by immunofluorescence. Cells grown on coverslips were fixed and immunostained with polyclonal anti-KIF23 V1 antibody, followed by detection with FITC-conjugated anti-rabbit IgG secondary antibody. **b** Subcellular localization of KIF23 V1 examined by cell fractionation and Western blotting. HLE cells were separated into nuclear and cytoplasmic fractions. Equal amounts of proteins were loaded onto SDS-PAGE gels, and the protein expression was analyzed with anti-KIF23 V1, anti-tubulin (cytoplasmic marker), or anti-lamin B1 (nuclear marker) antibodies

To determine the subcellular localization of KIF23 V1 protein in HCC cells, immunofluorescence and Western blotting assays were performed. Immunofluorescence assay with anti-KIF23 V1 antibody demonstrated that the endogenous KIF23 V1 was located in the nucleus in both HLE and Huh7 HCC cell lines (Fig. 3a). We further investigated KIF23 V1 localization in a cell fractionation assay by Western blotting. The cytoplasmic and nuclear extracts of HLE cells were immunoblotted with anti-KIF23 V1 antibody and the endogenous KIF23 V1 protein was strictly found in nuclear extracts (Fig. 3b).

### Expression of KIF23 protein in HCC tissues

**Fig. 4:**
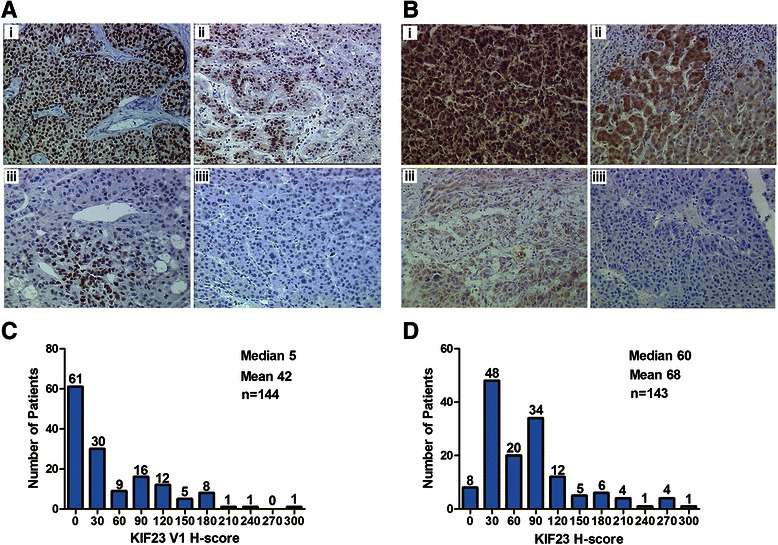
Expressions of KIF23 V1 and KIF23 V2 protein in HCC tissues. **a** Representative images of KIF23 V1 staining in HCC tissues. *i*: A strong stained HCC sample (*H*-score of 290). *ii*: A moderately stained HCC sample (*H*-score of 130). *iii*: A weakly stained HCC sample (*H*-score of 60). *iiii*: A negative stained HCC sample (*H*-score of 0). Magnification: 1:200. **b** Representative images of KIF23 V2 staining in HCC tissues. *i*: A strong stained HCC sample (*H*-score of 260). *ii*: A moderately stained HCC sample (*H*-score of 125). *iii*: A weakly stained HCC sample (*H*-score of 40). *iiii*: A negative stained HCC sample (*H*-score of 0). Magnification: 1:200. **c** Histograms showing the distribution and frequency for KIF23 V1 expression in HCC tissues. **d** Histograms showing the distribution and frequency for KIF23 V2 expression in HCC tissues

Expression of KIF23 protein in tumor cells was assessed by IHC with anti-KIF23 V1 or anti-KIF23 antibodies. When using anti-KIF23V1 antibody, we found that KIF23 V1 protein was mainly distributed in the nucleus of tumor cells, and all the tumor tissues displayed the heterogeneous pattern, with groups of tumor cells expressing very high level of KIF23 V1 protein, and others without any detectable expression. Examples of different expression levels of KIF23 V1 are depicted in Fig. 4a. KIF23 V1 protein was detectable in 83 of 144 (57.6 %) HCC tissues and the mean *H*-score was 42 (range: 0–290) (Fig. 4c). However, applying anti-KIF23 antibody for IHC staining, positive staining was predominantly observed in the cytoplasm of tumor cells, suggesting that KIF23 V2 localized in the cytoplasm of HCC cells. The tumor tissues also showed heterogeneous expression pattern for KIF23 V2, examples of different expression levels of KIF23 V2 protein are depicted in Fig. 4b. Cytoplasmic expression of KIF23 V2 was detected in 135 of 143 (94.4 %) HCC tissues and the median *H*-score was 60 (range: 0–290) (Fig. 4d).

For efficacy analyses, the population was divided into two groups according to the median *H*-score value. Expression level of KIF23 V2 was categorized as KIF23 V2 over-expressing tumors (>60) and KIF23 V2 low-expressing tumors (≤60). Because a small number of cases showed positive immunostaining, KIF23 V1 cases were classified into either negative or positive groups.

### Clinical relevance of KIF23 V1 expression in HCC tissues

**Fig. 5:**
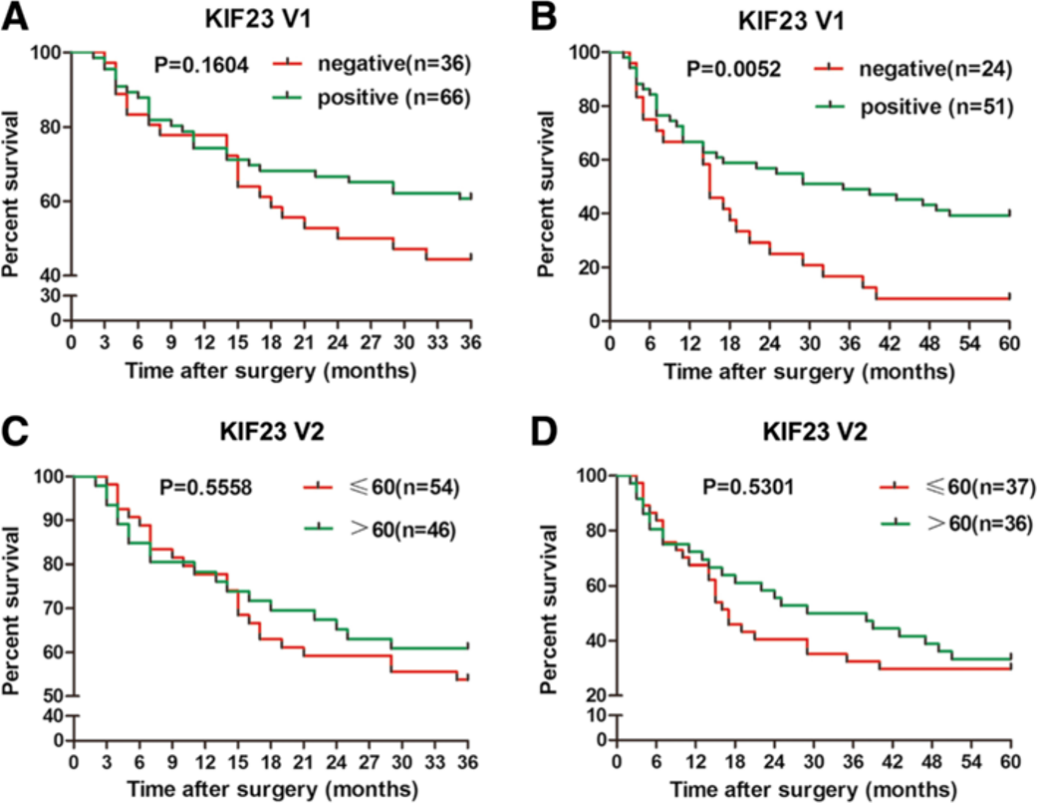
Association between KIF23 V1 or KIF23 V2 protein expression and overall survival (OS) of HCC patients. Correlation of KIF23 V1 expression with 3-year (**a**) or 5-year (**b**) OS of HCC patients. Correlation of KIF23 V2 expression with 3-year (**c**) or 5-year (**d**) OS of HCC patients. The group with expression of KIF23 V1 showed significantly better survival than the group without expression of KIF23 V1 (*p* = 0.0052). Patients were categorized by the median *H*-scores of KIF23 V2 (60) or by KIF23 V1 expression or not

The association between KIF23 V1 expression and overall survival (OS) was evaluated using Kaplan-Meier survival curves with the log-rank test. HCC patients with tumors expressing KIF23 V1 tended to correlate with better 3-year survival, but without statistical significance (*P* = 0.1604, Fig. 5a), while KIF23 V1-expressing patients had significantly longer OS (35 months) than the patients whose tumors did not express KIF23 V1 (15 months) (*P* = 0.0052, Fig. 5b). In addition, the association between the expression level of KIF23 V1 and clinical parameters was analyzed, and no correlation was observed between KIF23 V1 expression and gender, age, tumor size or TNM stage.

In addition, the association between the expression level of KIF23 V2 and clinical parameters was also analyzed. Based on the cut-off point of KIF23 V2, 67 patients were divided into a high expression group and 76 patients into a low expression group. No significant association between KIF23 V2 expression level and gender, age, tumor size or TNM stage was found. Furthermore, KIF23 V2 expression level did not associate with 3-year and 5-year OS (Fig. 5c, d).

In the univariate survival analysis, we correlated different parameters with the 5-year survival rate. KIF23 V1 expression (negative vs. positive; *P* = 0.0097), tumor size (≤5 cm vs. >5 cm; *P* = 0.0186), TNM stage (I, II vs. III, IV; *P* = 0.0040), were identified as parameters significantly influencing survival (Table 1). Multiple Cox regression analysis indicated that TNM stage was an independent prognostic predictor of the 5-year overall survival rates in HCC patient after surgery (Table 1).

**Table 1:** Prognostic factors for survival

Variable	OS		
	Univariate	Multivariate	
	*p*-value	HR (95 % CI)	*p*-value
Gender (Male versus Female)	NS		NA
Age (≤55 vs. >55)	NS		NA
Size (≤5 cm vs. >5 cm)	0.0186		NS
TNM stage (I, II vs. III, IV)	0.0040	1.502 (1.121-2.014)	0.0059
KIF23 V2 (≤60 vs. >60)	NS		NA
KIF23 V1 (negative vs. positive)	0.0097		NS

Univariate analysis: *χ*^2^ test

Multivariate analysis: Cox proportional hazards regression model

Abbreviations: *OS* Overall survival, *HR* Hazard Ratio, *CI* confidence interval, *TNM* tumor-node-metastasis, *NA* not adopted, *NS* not significant

## Discussion

In present study, the expression of the two splice variants of KIF23 mRNA was detected in most clinical HCC samples and cell lines. Using the prepared antibody specific to KIF23 V1, we found the distinct expression patterns of KIF23 V1 and V2 protein in HCC tumor tissues. Moreover, the expression of KIF23 V1 protein was associated with prolonged overall survival in the patients with HCC.

KIF23 is a member of kinesin-like motor protein families [20] and plays an important role in cytokinesis [9, 10, 21]. Two splice variants of KIF23 mRNA have been reported [16]. However, the differences in the localization, expression, and function for the two splice variants of KIF23 in tumor cells have remained largely unknown so far. No commercial antibodies are available for distinguishing KIF23 V1 from V2 at present. In the current study, we prepared anti-KIF23 V1 antibody, and confirmed the specificity of the antibody by overexpression of KIF23 V1 and V2 as well as knockdown of KIF23 V1. Immunofluorescence staining and cell fraction analysis with the prepared antibody specific to KIF23 V1, we found that endogenous KIF23 V1 was predominantly localized in the nucleus of the two HCC cell lines (HLE and Huh7), which was consistent with the previous report that CHO1 (KIF23 V1) isoform was present in the nucleus of CHO and HeLa cells [16].

Immunohistochemical staining of HCC tissues with anti-KIF23 V1 or anti-KIF23 antibodies indicated that tumor tissues were significant heterogeneity with some tumor cells expressing high levels of KIF23 V1 or V2 protein while being undetectable in others. Using the antibody specific to KIF23 V1 for immunohistochemical staining of HCC tissues, we also found that KIF23 V1 was predominantly localized in nucleus of tumor cells, which was quite different from the positive staining in cytoplasm using commercial anti-KIF23 antibody. The differential expression patterns for the two splice variants of KIF23 suggest that they may have distinct activities in tumor cells. We further found that the expression of KIF23 V1 protein was significantly associated with prolonged overall survival. The univariate Cox regression analysis revealed that KIF23 V1 expression is a factor that significantly influences the outcomes of HCC patients.

In this study, we observed a favorable effect of KIF23 V1 expression on overall survival. This finding is in contrast with our expectations that KIF23 might promote tumor development, as KIF23 V1 is upregulated in HCC tissues and previous report showed that downregulation of KIF23 decreases proliferation of glioma cells [15]. Furthermore, both KIF23 V1 and V2 have been recently shown to be down-regulated by tumor suppressor p53 in a p21-dependent pattern [22]. We speculate that KIF23 V1 may be involved in hepatocarcinogenesis, however, once the tumor is formed, it may play a negative role during the progression of cancer. Of course, in order to achieve a better understanding of the mechanism of KIF23 V1 expression in carcinogenesis and progression of cancer in patients with HCC, further studies on the biological functions of KIF23 V1 and V2 as well as their relationships in tumor cells are necessary.

## Conclusions

In conclusion, we prepared polyclonal antibody specific to KIF23 V1 to distinguish KIF23 V1 from KIF23 V2, and we show for the first time that KIF23 V1 and KIF23 V2 have different localizations in tumor cells. Furthermore, we found that both KIF23 V1 and KIF23 V2 are up-regulated in HCC patients, and KIF23 V1 expression might be a marker of longer overall survival in HCC patients.

## Abbreviations

CI: confidence interval
HR: hazard ratio
KIF23: kinesin family member 23
NA: not adopted
NS: not significant
OS: overall survival
PBMC: peripheral blood leukocyte
RT-PCR: reverse transcription-polymerase chain reaction
TNM: tumor- node- metastasis
